# Use of healthcare REsources and associated COsts in controlled versus uncontrolled carcinoid SYndrome in patients with neuroendocrine tumours: the RECOSY study

**DOI:** 10.1007/s12094-021-02608-7

**Published:** 2021-06-09

**Authors:** A. Custodio, P. Jimenez-Fonseca, A. Carmona-Bayonas, M. J. Gomez, M. I. Del Olmo-García, I. Lorenzo, J. Á. Díaz, N. Canal, G. De la Cruz, C. Villabona

**Affiliations:** 1 Medical Oncology Department, Hospital Universitario La Paz, IdiPAZ, CIBERONC CB16/12/00398, Madrid, Spain; 2 Medical Oncology Department, Hospital Universitario Central de Asturias, ISPA, Oviedo, Spain; 3 Haematology and Medical Oncology Department, Hospital Universitario Morales Meseguer, UMU, IMIB, CP13/00126, PI17/0050 (ISCII & FEDER), Murcia, Spain; 4 Fundación Séneca (04515/GERM/06), Murcia, Spain; 5 Medical Oncology Department, Hospital Universitario Puerta del Mar, Cádiz, Spain; 6 Medical Oncology Department, Hospital La Fe, Valencia, Spain; 7 Endocrinology Service, Complejo Hospitalario Universitario de Vigo, Pontevedra, Spain; 8 Endocrinology Service, Hospital Clínico de San Carlos, Madrid, Spain; 9 IQVIA, Barcelona, Spain; 10 Ipsen, Hospitalet de Llobregat, Barcelona, Spain; 11 Endocrinology Service, Hospital Bellvitge, Hospitalet de Llobregat, Barcelona, Spain

**Keywords:** Neuroendocrine tumour, Carcinoid syndrome, Resources, Costs, Somatostatin analogues

## Abstract

**Purpose:**

To report healthcare resource use and associated costs in controlled versus uncontrolled carcinoid syndrome (CS) in patients with neuroendocrine tumours.

**Methods:**

A cross-sectional, non-interventional multicentre study was conducted with retrospective data analysis. Resource use was compared between two patient groups: those with controlled CS (> 12 months with no uncontrolled CS episodes) and uncontrolled CS (< 12 months since last uncontrolled episode). Patients were matched for age, sex, and origin and grade of tumour. When no matching patients were available, data from deceased patients were used. Information on healthcare resource use came from review of medical records, patient history and physician reports. Working capacity was assessed using the Work Productivity and Activity Impairment General Health questionnaire.

**Results:**

Twenty-six university hospitals in Spain participated, between July 2017 and April 2018. 137 patients were enrolled; 104 were analysed (2 groups of 52). Patients with uncontrolled CS had 10 times more emergency department (ED) visits (mean 1.0 vs 0.10 visits; *P* = 0.0167), were more likely to have a hospital admission (40.4% vs 19.2%; *P* = 0.0116) and had longer hospital stays (mean 7.87 vs 2.10 days; *P* = 0.0178) than those with controlled CS. This corresponded to higher annual hospitalisation costs (mean €5511.59 vs €1457.22; *P* = 0.028) and ED costs (€161.25 vs €14.85; *P* = 0.0236). The mean annual total healthcare costs were 60.0% higher in patients with uncontrolled than controlled CS (*P* = NS).

**Conclusion:**

This study quantifies higher health resource use, and higher hospitalisation and ED costs in patients with uncontrolled CS. Better control of CS may result 3in lower medical costs.

**Supplementary Information:**

The online version contains supplementary material available at 10.1007/s12094-021-02608-7.

## Introduction

Neuroendocrine tumours (NETs) are a diverse group of tumours derived from neural ridge cells, endocrine glands, islets or cells, which share the propensity to secrete active hormones such as serotonin [1]. Although they are relatively rare, with an incidence of around 6.98/100,000/year and accounting for 0.5% of all newly diagnosed malignancies [2], their incidence has markedly increased over the past 15 years [3, 4]. While this increased incidence may be due to improved detection [3], diagnosis remains complex. This is in part due to the nonspecific and often late-presenting symptoms: many patients attend several specialists and undergo multiple investigations before a definitive diagnosis is made [1]. Diagnosis at a late stage also reduces the likelihood of being able to perform a complete surgical resection [5], and advanced NETs are frequently managed as chronic conditions, with long-term and often multiple treatments required [6].

Carcinoid syndrome (CS) occurs in around 10% of patients with NETs [7]. Its incidence varies with tumour type [8], increasing with tumour bulk and disease severity, such that it occurs in up to 30% of patients with well-differentiated tumours [9]. A recent large, population-based study found CS was present in up to 19% of patients with NETs [10]. The most common symptoms of CS are diarrhoea, flushing, and dyspnoea (bronchospasm), which, due to disease progression or resistance to somatostatin analogues (SSAs), can require treatment escalation and modification. With disease progression, mesenteric or retroperitoneal fibrosis may occur, as well as carcinoid heart disease. Occasionally, following a trigger such as general anaesthetic, a severe and life-threatening carcinoid crisis may occur, with massive uncontrolled release of amines, resulting in arrhythmias and cardiovascular instability [11].

The symptoms of CS are thought to be mainly due to serotonin, although histamine, kallikrein, prostaglandins, and tachykinins are likely to play a role. SSAs inhibit the secretion of serotonin and other neuropeptides [12]. Current guidelines recommend SSAs as standard therapy in functional NETs of any origin [13], and they (octreotide and lanreotide) are an effective treatment in 70–90% of patients with CS [7, 13–16].

NETs and CS adversely impact quality of life, affecting physical and mental health (depression, anxiety), social aspects, and ability to work [1, 17–19]. Some studies in recent years have assessed the economic effects of NETs, CS, or prevalent CS symptoms, mainly in US populations [20–22]. One study looked at the use of healthcare resources and associated costs in a European setting [23]. Given this scarcity of data from a European population, we sought to quantify the resource use and the economic implications of uncontrolled CS at a national level within Spain. Taking into account the profound effect of CS on quality of life, and its chronic progressive nature, we hypothesised that patients with uncontrolled CS would have higher use of healthcare resources and consequently incur higher associated costs than those with controlled CS.

## Methods

### Study design and patients

This was a non-interventional, cross-sectional, observational, multicentre study with retrospective data analysis, conducted in outpatient endocrinology and medical oncology departments.

Eligible patients were ≥ 18 years old with metastatic grade 1–2 NETs and CS treated with SSAs. Patients were categorised as having *uncontrolled* or *controlled CS* based on the presence or absence of uncontrolled episodes within the previous 12 months. An *uncontrolled CS episode* was defined as at least one of the following: > 4 episodes of diarrhoea per day for > 1 month, > 1 month of flushing (≥ 1 flushing episode/week for > 1 month), doses of SSAs higher than the standard doses (> 120 mg/4 weeks of lanreotide or > 30 mg/4 weeks of octreotide), or worsening of carcinoid heart disease or mesenteric fibrosis. The study excluded patients with other malignant disease, or with other severe diseases that could interfere with assessment of CS, such as right cardiomyopathy or diarrhoea of a different aetiology, those participating in another clinical study, and those who could not read or understand the study questionnaires.

The study aimed to enrol 130 patients in total, divided into the *controlled CS* and *uncontrolled CS* groups. Patients in these two groups were matched for tumour origin and grade. Where possible, they were also matched for evidence of stability/progression and length of treatment (< 1 year; 1–2 years; or > 2 years). If insufficient matched patients were available, then deceased ones were included, provided their medical records contained the minimum information required.

The study consisted of a single visit that coincided with an already-scheduled medical appointment, during which sociodemographic and clinical variables were collected directly from the patients or from their medical records. The physician provided information on the clinical status of the disease and the patients completed a questionnaire on their working capacity. Data were collected from 6 to 12 months before the study visit for patients with controlled CS, and from 3 months before and 6 to 12 months after the last CS episode for patients with uncontrolled CS. The analysis was adjusted according to the relative time at which the information was collected: from 6 to 12 months in the uncontrolled vs from 9 to 15 months in the controlled CS group.

### Objectives and variables

The primary objective was to determine the use of healthcare resources and their associated costs and the effects on capacity to work in patients with controlled and uncontrolled CS and grade 1–2 metastatic NETs receiving SSAs. Direct costs comprised number of hospitalisations, length of stay, visits to emergency departments (ED), outpatient visits including primary care, investigations performed, treatment received for NET and CS, and surgical and other procedures related to NET or CS.

Effects on capacity to work were based on the responses to the Work Productivity and Activity Impairment General Health (WPAI-GH) questionnaire [24], which assesses the occupational impact of a disease over the past 7 days. Only those patients in employment at the time of the study visit completed this questionnaire. From the responses, we calculated *absenteeism* (number of hours lost/total number of hours the individual should have worked); *presenteeism,* defined as deterioration in the capacity to work, rated on a Likert-type scale from 0 (no deterioration) to 10 (completely unable to perform tasks); *overall work impairment* and *activity impairment* due to health, all expressed as percentages [25]. In addition, patients with uncontrolled CS answered ad hoc questions on occupational absenteeism at the time of the last carcinoid episode, and deceased patients’ medical records were reviewed to answer as many as possible of the ad hoc questions. Supplementary materials S1 and S2 contain the WPAI-GH questionnaire and the ad hoc questions.

The secondary objective was to describe the clinical characteristics and management of the patient groups. The following variables were collected: NET diagnostic procedure, time since NET diagnosis, origin and grade of tumour, treatment prescribed following NET diagnosis, time from diagnosis to first intervention, NET treatment, CS treatment, current symptoms, comorbidities, New York Heart Association (NYHA) classification, pro-brain natriuretic peptide (proBNP) levels, chromogranin A and 5-hydroxyindoleacetic acid (5-HIAA) levels, latest echocardiogram result, Eastern Cooperative Oncology Group (ECOG) performance status, patient-perceived health status and physician-reported disease status. In patients with uncontrolled CS, the time since the last carcinoid episode and the duration of the episode were also described, and the number and percentage of patients with valvular heart disease were calculated. Physicians and patients were also asked to categorise their (patients’) health status as *good*, *neither good nor bad*, or *bad,* to determine whether their opinions correlated.

### Ethics

This non-interventional study was approved by the Ethics Committee of *Hospital Universitari de Bellvitge,* Barcelona (27 April 2017), and classified as a post-approval study by the Spanish Agency for Medicines and Medical Devices (AEMPS; 16 February 2017). The study followed the International Society for Pharmacoepidemiology Guidelines on Good Pharmacoepidemiological Practice [26]. Prior to any data collection, living participants received a full explanation and provided written informed consent. In the case of deceased participants, following ethics committee review, consent from living relatives was not required.

### Statistical analysis

Unmatched patients were excluded from all analyses. Use of healthcare resources (hospitalisation, hospital department and length of stay, visits to ED, outpatient and primary care visits, tests and medical interventions performed) were analysed using descriptive statistics. The two groups (controlled vs uncontrolled CS) were then compared using the Cochran–Mantel–Haenszel test for resource consumption (any use) and paired *t* test for the number of times the resource was used. Direct costs were calculated per patient and per year, based on local costs per unit. Data on costs were obtained from national data sources [27, 28], according to the perspective of the Spanish national health system. Direct costs between groups were compared using the paired *t* test and the Wilcoxon test.

The secondary endpoints of clinical characteristics and management were analysed using descriptive analysis and compared between groups using a paired *t* test for continuous variables and the Cochran–Mantel–Haenszel test or conditional logistic regression for categorical variables.

Given the lack of existing comparative data on patients with controlled vs uncontrolled CS, the study sample size was calculated using a continuous variable. To detect differences ≥ 0.35 standard deviation (moderate differences) between patients with controlled or uncontrolled CS (paired data) with a significance level of 0.05 and a statistical power of 0.8, a sample size of ≥ 65 patients per group was determined. All data were collected from real clinical practice; if an assessment was not performed as part of routine care, missing data were not replaced.

## Results

### Participants

Twenty-six university hospitals in Spain participated, between July 2017 and April 2018. One hundred and thirty-seven patients were enrolled; 33 were excluded (no matching patient [24], not treated with an SSA [6], or taking part in another study [3]), leaving 104 for the analysis, 52 in each group. The uncontrolled CS group included 10 (19.2%) deceased patients. Overall, the mean age was 64.3 years and 53.8% were men. The sociodemographic and clinical characteristics of the two groups are shown in Table 1. Clinical and treatment characteristics are reported below as secondary outcomes.

**Table 1:** Patient demographics

Variable	Uncontrolled CS	Controlled CS
Age in years, mean (SD); min–max	63.75 (12.17); 37–87	64.88 (12.04); 38–92
Sex, female, *n* (%)	22 (42.3%)	26 (50.0%)
Employment status, *n* (%)	*n* = 42^b^	*n* = 52
Employed	6 (14.3%)	10 (19.2%)
Unemployed	6 (14.3%)	14 (26.9%)
Other^a^	30 (71.4%)	28 (53.8%)
Education level	*n* = 52	*n* = 52
No formal education	4 (7.7%)	2 (3.8%)
Primary	20 (38.5%)	20 (38.5%)
Secondary	15 (28.8%)	18 (34.6%)
University or postgraduate studies	13 (25.0%)	12 (23.1%)

*SD* standard deviation, *min* minimum, *max* maximum

^a^Other: students, retired, homemaker

^b^Data not available in ten patients (not recorded in notes)

### Primary objective: healthcare resource use and direct associated costs

#### Hospitalisation, ED visits and outpatient clinics

There were large and statistically significant differences in acute medical care between the two groups (Table 2). In the uncontrolled CS group, 40.4% of patients required hospital admission vs 19.2% of the controlled CS group (*P* = 0.0116), and the duration of hospital stay was longer in the uncontrolled (mean 7.9 days) than in the controlled (mean 2.1 days) CS group (*P* = 0.0178). More patients in the uncontrolled (32.7%) than in the controlled CS group (5.8%) visited the ED (*P* = 0.0010), and the mean number of ED visits was ten times higher in the uncontrolled CS group than in the controlled CS group (1.0 vs 0.10, respectively; *P* = 0.0167).

**Table 2:** Hospital admissions, emergency department visits, and outpatient clinic visits during the study period and their corresponding annual costs in Euros

	Usage during study period^a^			Annual cost, euros, mean (SD)		
	Uncontrolled CS *n* = 52	Controlled CS *n* = 52	*P *value	Uncontrolled CS	Controlled CS	*P* value
Any hospitalisation, *n* (%)	21 (40.4%)	10 (19.2%)	0.0116*^b^	5511.59 (10,166.14)	1457.22 (3346.59)	0.0280*^c^
No. of hospitalisations, mean (SD)	0.62 (0.97)	0.25 (0.59)	0.0024*^c^			
Length of stay, days, mean (SD)	7.87 (13.66)	2.10 (4.90)	0.0178*^c^			
Department admitted to, *n* (%)						
General and GI surgery	5 (9.6%)	5 (9.6%)				
Endocrinology	4 (7.7%)	0				
Internal medicine	2 (3.8%)	0				
Nuclear medicine	1 (1.9%)	1 (1.9%)				
Medical oncology	13 (25.0%)	3 (5.8%)				
Palliative care	1 (1.9%)	0				
ICU	1 (1.9%)	0				
GI	0	1 (1.9%)				
Any ED visit, *n* (%)	17 (32.7%)	3 (5.8%)	0.0010*^b^	161.25 (333.48)	14.85 (63.13)	0.0236*^c^
No. of ED visits, mean (SD)	1.0 (2.05)	0.10 (0.41)	0.0167*^c^			
Any outpatient visit, *n* (%)	32 (61.5%)	33 (63.5%)	0.8273^b^	633.34 (838.32)	510.03 (661.29)	0.2322^c^
No. of outpatient visits (all specialties), mean (SD)	6.48 (8.41)	5.87 (7.54)	0.1731^c^			
No. of outpatient visits, by specialty, mean (SD)						
Primary care	0.37 (2.11)	0.58 (2.86)				
Cardiology	0.38 (0.75)	0.13 (0.34)				
General surgery	0.44 (2.04)	0.79 (2.27)				
Dermatology	0.04 (0.28)	0.00 (0.00)				
Endocrinology	1.10 (2.56)	0.85 (1.66)				
Gynaecology	0 (0.00)	0 (0.00)				
Internal medicine	0 (0.00)	0.02 (0.14)				
Nuclear medicine	0.15 (0.64)	0.23 (0.85)				
Pneumonology	0.10 (0.41)	0 (0.00)				
Medical oncology	3.85 (5.85)	3.15 (5.15)				
Interventional radiology	0.02 (0.14)	0.00 (0.00)				
Nephrology	0 (0.00)	0.02 (0.14)				
Neurology	0 (0.00)	0.04 (0.28)				
Ophthalmology	0 (0.00)	0.04 (0.19)				
Radiation oncology	0.04 (0.28)	0.02 (0.14)				

*ED* emergency department, *GI* gastrointestinal, *ICU* intensive care unit, *SD* standard deviation

*Statistically significant (*P* < 0.05)

^a^Adjusted according to the relative time at which the information was collected: from 6 to 12 months in the uncontrolled vs from 9 to 15 months in the controlled CS group

^b^Mantel–Haenszel Chi-square

^c^Paired *t* test

There were no statistically significant differences in total outpatient attendances between the two groups: 61.5% of the uncontrolled CS group and 63.5% of the controlled CS group had at least one visit, and the mean number of visits was 6.2 overall. Table 2 includes breakdown by department and specialty for ED and outpatient attendance.

This direct healthcare usage translated to a statistically significant difference in the annual cost of hospitalisation for uncontrolled vs controlled CS: mean €5511.59 vs €1457.22, respectively (*P* = 0.0280) (Table 2). Mean ED costs were also significantly higher in uncontrolled than controlled CS: €161.25 vs €14.85 per patient year (*P* = 0.0236). Total outpatient clinic costs were higher in patients with uncontrolled vs controlled CS, without reaching statistical significance. Visits to the medical oncologist constituted the highest outpatient costs (*P* = NS); this was followed by endocrinology visits, which were more frequent in patients with uncontrolled CS (*P* = 0.0350).

#### Investigations: imaging, laboratory tests and biopsies

Overall, 97.1% of patients had undergone at least one imaging study (morphological or functional) during the period assessed: mean (SD) 4.2 (2.8) tests per patient. The most-used techniques were high-resolution computed tomography (HRCT) (in 90.4%), scintigraphy with octreotide (59.6%), magnetic resonance imaging (28.8%), and abdominal ultrasound (20.2%). There were no significant differences between the two groups for any of these investigations.

Abdominal X-ray was performed in 5 patients with uncontrolled CS and in 0 patients with controlled CS (*P* = 0.0253). Overall, 9.6% of patients underwent endoscopy, but there were no significant differences between the two groups. Table 3 shows the number of tests performed per group.

**Table 3:** Use of investigations by category during the study period and corresponding annual costs in Euros

Investigation	Usage during study period, no. performed			Annual cost, euros		
	Uncontrolled CS *n* = 52	Controlled CS *n* = 52	*P* value	Uncontrolled CS	Controlled CS	*P* value
Morphological and functional imaging	4.69 (3.27)	3.63 (2.11)	0.3273^a^	961.60 (931.03)	887.41 (907.57)	0.2317^a^
Endoscopy	0.13 (0.40)	0.08 (0.27)	0.7845^a^	87.96 (282.67)	49.67 (173.74)	0.8803^a^
Laboratory tests	16.71 (11.61)	10.85 (7.86)	0.0730^a^	1967.86 (1721.73)	1257.45 (1056.27)	0.1302^a^
*Blood tests*	14.44 (10.79)	9.40 (7.15)	0.1502 ^a^	–	–	–
*Urine tests*	2.27 (2.47)	1.44 (1.67)	0.0084^a^	–	–	–
Biopsy	0.46 (1.24)	0.17 (0.43)	0.0744^a^	560.11 (1643.71)	91.73 (228.05)	0.0981^a^
Immunohistochemistry	0.58 (1.21)	0.50 (1.28)	0.4402^a^	28.48 (60.71)	23.80 (60.71)	0.4775^a^
Total investigations				3606.01 (3133.90)	2310.06 (1829.55)	0.0486*^a^

Mean (SD) for all parameters. Further breakdown within categories is provided in Supplementary Table S3

*CS* carcinoid syndrome

*Statistically significant (*P* < 0.05)

^a^Paired *t* test

Overall, 95.2% of patients had at least one laboratory test (e.g. blood or urine). There was a significant difference in the mean number of urine tests performed: 2.27 in the uncontrolled vs 1.44 in the controlled CS group (*P* = 0.0084), but not in the mean number of blood tests.

There were no significant differences between groups in the number of patients undergoing biopsy or the number of biopsies performed. Immunohistochemistry staining (which included chromogranin, synaptophysin, enolase, insulin, CD56, CDX2, KI67, PAX8, and TTF-1) was also not significantly different.

The annual cost of all investigations was significantly higher in the uncontrolled CS group than in the controlled CS one: mean €3606 vs €2310 (*P* = 0.0486). Table 3 shows investigation costs by category, and supplementary material S3 shows further breakdown of usage within each category.

#### Treatments

##### Pharmacological treatment for CS

The mean total annual cost of pharmacological treatment for CS was higher in the uncontrolled CS (€25,924.80) than in the controlled CS group (€18,219.15) but the difference did not reach statistical significance (*P* = 0.2228) (Table 4). Pharmacological treatment represented 80.2% of patients with NETs’ total annual cost. As part of the study inclusion criteria, all participants were receiving SSAs, and they made up 48.7% of the annual treatment costs. In patients with uncontrolled CS, SSA costs made up 37.0% of the mean total pharmacological treatment costs, while in patients with uncontrolled CS, they made up 58.8%.

**Table 4:** Treatment usage, corresponding annual treatment costs, and total annual healthcare costs in Euros

Treatment	Usage, number (%) of patients on this treatment			Costs, mean (SD)			
	Uncontrolled CS (*n* = 52)	Controlled CS (*n* = 52)	*P* value	Uncontrolled CS	Controlled CS		*P* value
All pharmacological treatments				25,924.70 (29,134.79)	18,219.15 (16,872.26)		0.2228
NET therapies							
Somatostatin analogues	52 (100%)	52 (100%)	na	10,780.83 (6470.63)	10,721.55 (5929.63)		0.9285
Chemotherapy	3 (5.8%)	2 (3.8%)	0.6547	25.03 (105.57)	163.39 (1119.24)		0.3805
Targeted therapy	18 (34.6%)	9 (17.3%)	0.0495*	10,710.17 (23,236.10)	5437.10 (15,076.08)		0.2863
Other	4 (7.7%)	3 (5.8%)	0.7055	3527.39 (14,244.52)	1897.12 (9220.04)		0.5505
CS therapies							
Antiemetics	2 (3.8%)	na	na	11.85 (62.50)	na		na
Antidiarrheals	17 (32.7%)	na	na	88.54 (201.05)	na		na
Antihistamines	2 (3.8%)	na	na	0.21 (1.18)	na		na
Other	5 (9.6%)	na	na	780.69 (3527.40)	na		na
Interventions and surgical procedures				3548.50 (11,905.15)		2032.21 (8292.85)	0.8885
Chemoembolization	1 (1.9%)	1 (1.9%)					
Radioembolization	1 (1.9%)	1 (1.9%)					
Liver transplant	1 (1.9%)	0					
Partial liver resection	1 (1.9%)	3 (5.8%)					
Right hemicolectomy and partial resection of omentum	1 (1.9%)	0					
Tricuspid and pulmonary valve replacement	1 (1.9%)	0					
Change of biliary prosthesis	0	1 (1.9%)					
Ileal resection	0	2 (3.8%)					
Small bowel resection	0	1 (1.9%)					
Lutetium therapy	1 (1.9%)	0					
Total annual direct costs (treatment + investigations + clinical care)							

	Uncontrolled CS	Controlled CS	*P* value
Mean (SD)	39,385.39 (32,438.53)	24,543.50 (19,449.49)	0.0535
Median (IQR)	25,708.50 (15,816.70–64,756.20)	18,382.50 (14,659.20–25,688.40)	
Min–Max	3172.00–145,678.40	2036.60–82,946.30	

*CS* carcinoid syndrome, *IQR* interquartile range, *NET* neuroendocrine tumour, *na* not available, *max* maximum, *min* minimum, *SD* standard deviation

##### Interventions and surgical procedures (related to NETs)

There was no difference in the overall use of interventions between the two groups. Seven out of 52 patients in each group underwent an intervention (e.g. chemo-/radioembolization, valve replacement or bowel resection). Table 4 shows the breakdown of procedures performed and corresponding annual costs.

##### Total direct costs

The mean annual direct healthcare costs, that is, investigations plus treatments plus inpatient and outpatient medical care, were 60% higher in patients with uncontrolled CS than patients with controlled CS, though this difference did not reach statistical significance: mean (SD) €39,385.39 (€32,438.53) in patients with uncontrolled CS vs €24,543.53 (€19,449.49) in patients with controlled CS (*P* = 0.0535) (Table 4).

### Effect on capacity to work: WPAI-GH questionnaire and ad hoc questions

Of the 17 patients who were employed at the study visit, 12 completed the WPAI-GH questionnaire. The questionnaire was not completed for deceased patients. There were insufficient paired cases to perform a comparison between both groups. Table 5 reports the WPAI-GH results by group. Of note, there was 32.5% absenteeism in the uncontrolled CS group vs 20.5% in the controlled CS group.

**Table 5:** Results of the WPAI-GH questionnaire

Variable	Uncontrolled CS	Controlled CS
Absenteeism, %	32.5 (47.2)	20.5 (36.5)
Presenteeism, %	26.7 (46.2)	22.9 (29.8)
Overall work impairment, %	28.7 (49.7)	27.6 (33.9)
Activity impairment, %	30.0 (52.0)	24.3 (32.6)

Results are reported as mean percentage (standard deviation) per patient

Forty-one patients from the uncontrolled CS group completed the ad hoc questions. These showed that at the time of the last carcinoid episode only two patients were in employment. Of the 39 who were not in employment, 21/39 (53.8%) were retired, 12/39 (31.6%) were on medical leave and 5/39 (12.8%) were unemployed (missing data on one patient). Of the two patients in employment, neither had medical leave during their last CS episode, but one patient had had to leave work early (any incidence) and one had been unable to go to work (any incidence).

### Secondary objectives: clinical characteristics and management

Supplementary material S4 contains the full details of the clinical characteristics of the patients in both groups; the groups were well matched. Overall, the median [interquartile range (IQR)] time between NET diagnosis and the study visit was 4.9 (2.2–8.5) years. The most frequent (73.1%) tumour origin was intestine, and 53.8% of NETs were grade 1. The most common site of metastases was the liver. NETs were most frequently diagnosed with morphological imaging (77.9%); HRCT in 70.2%. The most common tumour studies for diagnosis were chromogranin A and synaptophysin; serum chromogranin A and urinary 5-HIAA were also used frequently (supplementary material S4). The most common first therapeutic intervention was surgery, in 62.5% (43.3% had partial resection of primary tumour); 37.5% received pharmacological treatment as their first treatment. The median time between diagnosis and first intervention was 18.5 days (IQR 0.0–57.7 days). As expected, the most commonly reported symptoms were diarrhoea, skin flushing, and abdominal pain. Only four patients in the uncontrolled CS group reported dyspnoea. Overall, 55.8% of patients had comorbidities, the most common being hypertension (31.7%), hyperlipidaemia (15.4%) and diabetes mellitus (11.5%).

Supplementary material S4 also contains the mean values for biochemistry markers and echocardiography results by group. Overall, 57.9% of patients had a normal echocardiogram while 28.1% had tricuspid valve disease. According to NYHA classification, 8.7% of patients with uncontrolled CS has moderate or severe (class III or IV) limitation of physical activity, while no patient in the controlled CS group had this degree of limitation (*P* = 0.0455). In total, 27.7% of the patients included in this study had an ECOG performance status of 0, and 94.7% had ECOG of 0 or 1. Statistically significant differences in ECOG were not observed between groups.

**Fig. 1:**
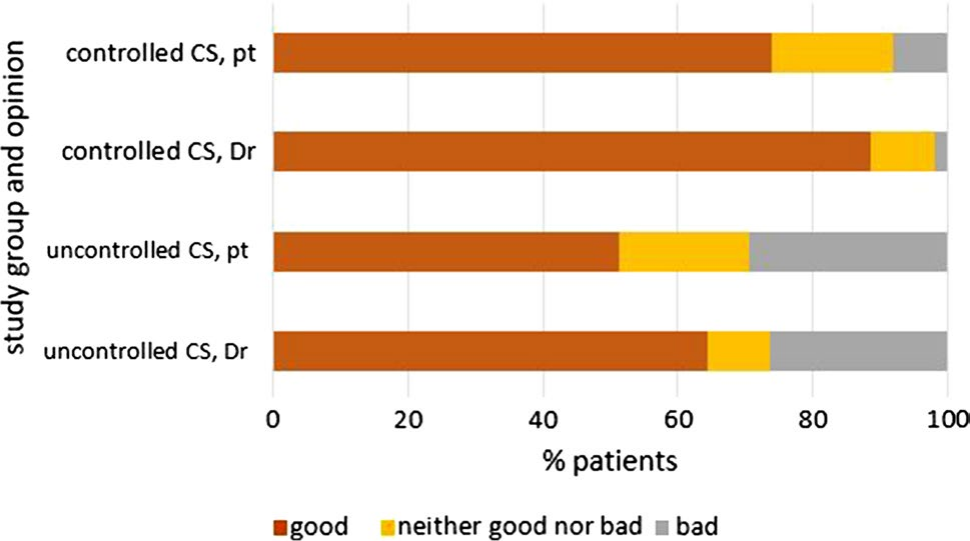
Perceived health status (good, neither good nor bad, or bad) according to patients and their physicians. *CS* carcinoid syndrome, *Dr* physician’s opinion; *pt* patient’s opinion

Figure 1 shows the perceived health status according to the physician or patient perspective for the controlled and uncontrolled CS groups: physician and patient opinions were generally in agreement.

## Discussion

Our study aimed to quantify the use of resources and the associated costs in patients with grade 1–2 metastatic NETs, comparing those with controlled and uncontrolled CS. As a secondary objective we described the patients’ clinical characteristics and management. We found that patients with uncontrolled CS had significantly higher rates of acute healthcare resource use than patients with controlled CS: they were more likely to require hospitalisation, had longer duration of hospital stay, and incurred higher mean hospitalisation costs. In addition, patients with uncontrolled CS were more likely to visit the ED and had 10 times more ED visits, with consequent higher costs than patients with controlled CS.

Besides these acute care costs, costs in other areas such as outpatient clinic attendance, treatments, or investigations, while being higher in the uncontrolled CS group, did not reach statistical significance. Of the treatment costs, SSA costs were similar between groups (mean €10,780.83 and €10,721.55), whereas the mean cost of targeted therapies was close to double in the uncontrolled CS vs controlled CS group (€10,710.17 vs €5437.10). This could reflect the increased use of targeted therapy (e.g. everolimus) in an attempt to control symptoms, since this agent has been shown to improve symptoms when combined with SSAs [29].

Our findings of a 60.0% increase in total costs in patients with uncontrolled CS over a 12-month period are similar to those from a Swedish study by Lesén et al. [23], who described a close to 40% increase in total costs in those with uncontrolled vs controlled CS, for an 8-month period. Lesén et al. also found that patients with carcinoid heart disease incurred additional costs. We did not assess this cost specifically, although we did find a greater incidence of valvulopathy, and worse NYHA classification in the uncontrolled vs controlled CS group. Having more patients with valvulopathy in the uncontrolled CS group may be one of the underlying factors for the increased costs observed, due to additional investigations and outpatient clinic attendance. Carcinoid heart disease is an established complication of uncontrolled CS and, as such, should be considered as a potential cost burden within these patients. In the study by Lesén et al., differences were driven by SSA costs, medical interventions such as hepatic artery embolisation were higher, and there were no differences in hospitalisations or targeted therapies. This may reflect a lack of standardisation in how to manage patients with uncontrolled CS, probably due to lack of approved or evidence-based treatments in cases when SSAs alone do not control symptoms.

Regarding the effects of the disease on the capacity to work, it is difficult to draw clear conclusions about the impact of uncontrolled and controlled CS. A recent study by Singh et al. [1] found that 39% of patients with NETs (mean age, 56.8 years) were in employment, and 18% had a self-identified medical disability. In our study, the percentage of unemployed patients (21.3%) and patients with the employment status other (61.7%, *n* = 58) (including homemakers, students, and retirees), meant only 12 patients (11.8%) completed the WPAI-GH questionnaire. Based on the low percentage of employed patients who completed the questionnaire, it could be concluded that CS negatively affects the working situation of both study groups (controlled and uncontrolled CS), with a total absenteeism of 24.5% (higher in the uncontrolled CS group, 32.5% vs 20.5% in the controlled CS group), and a total presenteeism of 24% (26.7% in uncontrolled vs 22.9% in controlled CS group). However, the mean age of study participants (64.3 years) could go some way to explaining the large proportion with *other* status, as many of these individuals may be retired. The lack of distinction between different statuses in the category *other* could therefore be considered a limitation of the study. Nonetheless, the fact that at the time of the last carcinoid episode, only two patients in the uncontrolled group were actively working and 31.6% of the remaining patients were on medical leave also indicates a potential effect of uncontrolled CS on patients’ capacity to work. A more in-depth study on effects on capacity to work and corresponding financial costs would be beneficial to give a more global view of the costs incurred.

The study by Singh et al. also found that oncologists and haematologists made up 70% of outpatient clinic visits in patients with NETs [1]. In our study in the setting of metastatic NETs, medical oncology was also the most-visited specialty, which fits with expectations. Likewise, medical oncology was also the most frequent specialty under which patients were admitted to hospital. Although we did not differentiate between hospitalisation due to CS or to other causes, there were no major differences in comorbidities between the two groups. Therefore, given the similar levels of comorbidities and the specialties the patients were treated under, it would be reasonable to interpret that the increased costs could have derived from CS.

Few European studies have looked at resource use in CS. In 2017, Shen et al. [20] published a US study based on the SEER registry and Medicare data from 6700 patients, in which they analysed cost patterns in elderly NET patients, comparing those with and without CS. They found median total costs of more than 1000 USD/month higher, and significantly higher total inpatient and outpatient costs in patients with CS than those without CS, even after controlling for clinical and demographic factors. Patients with CS were significantly more likely to have ED attendance and hospitalisation. Our study design differed in that it did not look specifically at older patients (although mean age was 63.4 years) and in that we differentiated between controlled vs uncontrolled CS rather than the presence or absence of CS. Nonetheless, many of the patterns described by Shen et al. were reflected in our study, to a slightly more modest level, with a median increase in the uncontrolled CS group for total healthcare costs of over €7000 per year, and a mean increase of over €14,000.

Another US study [21], looking specifically at diarrhoea in CS, found that patients with diarrhoea had significantly higher rates of healthcare resource use than those without. In line with our findings, they also reported higher rates of hospitalisation, ED attendances, outpatient clinic visits and duration of hospital stay. CS-associated diarrhoea accounted for a 1.5-fold higher total healthcare spending, only slightly lower than our result of 60.0% (that is, 1.6-fold) higher costs in those with uncontrolled CS, which included any CS-related symptom (not only diarrhoea).

Huynh et al. [22], in an economic study on dose escalation of SSAs, found that patients with CS symptom improvement had significantly lower annual healthcare costs (14,766 USD per patient) than patients with continued symptoms of flushing or diarrhoea. Their study included an analysis of indirect costs, including, for example, hours spent at clinic visits and corresponding loss of earnings, a point which we did not calculate in the present study, but which would be informative.

Some limitations to the RECOSY study should be noted. First, this study was cross-sectional and based on medical records, patient history, and physician reporting. Therefore, any reported outcomes are likely to be subject to recall bias, and parameters that were not recorded as part of routine clinical practice were unavailable. However, strengths of the study include that these findings are reflective of real clinical practice, as the study was conducted in 26 public university hospitals throughout Spain, and included data from primary care. This characteristic, in addition to the fact that the Spanish public health system is universal, increases the likelihood of the findings being representative of the national population with NETs and CS. A second limitation is that, due to a lack of matching patients, deceased patients’ medical records had to be used, which is suboptimal; however, ensuring this matching we aimed to increase the robustness of the analysis.

To our knowledge, RECOSY is the first study to report the use of resources and the associated direct and indirect costs for CS in Spain, and is among the few that exist for European countries [23, 30]. It provides detailed data on the scope of the healthcare and economic burden of CS in this setting. Given that the incidence of NETs continues to rise and survival is increasing [2], the economic implications of NETs and CS management may proportionally increase further in the future. Our study should be considered alongside previous studies on quality of life [18, 19, 31, 32] to ensure that medical teams are aware of the impact of CS on patients’ lives and the resources they require, and how these factors can vary according to whether or not CS is controlled. It is hoped that this will be useful for those involved in research on this disease, and ultimately help in decision-making for appropriate allocation of health resources.

## Conclusion

Compared with patients with controlled CS, patients with uncontrolled CS require more ED visits, more hospital admissions, and longer hospital stays, resulting in higher acute care costs and a trend towards higher total costs.

## Supplementary Information

Below is the link to the electronic supplementary material.

Supplementary file1 (DOCX 117 KB)

## Declarations

### Availability of data and material

Where patient data can be anonymised, Ipsen will share all individual participant data that underlie the results reported in this article with qualified researchers who provide a valid research question. Study documents, such as the study protocol and clinical study report, are not always available. Proposals should be submitted to DataSharing@Ipsen.com and will be assessed by a scientific review board. Data are available beginning 6 months and ending 5 years after publication; after this time, only raw data may be available.
