# Supplementary material for: Use of healthcare REsources and associated COsts in controlled versus uncontrolled carcinoid SYndrome in patients with neuroendocrine tumours: the RECOSY study
Source: Clin Transl Oncol. 2021 Jun 9;23(10):2046–56. doi: 10.1007/s12094-021-02608-7 (PMC8390421; doi:10.1007/s12094-021-02608-7)
Supplement: Supplementary file 1 — Supplementary file1 (DOCX 117 KB) [file 12094_2021_2608_MOESM1_ESM.docx]

**Supplementary material**

**S1**: WPAI-GH questionnaire, used to assess indirect costs.


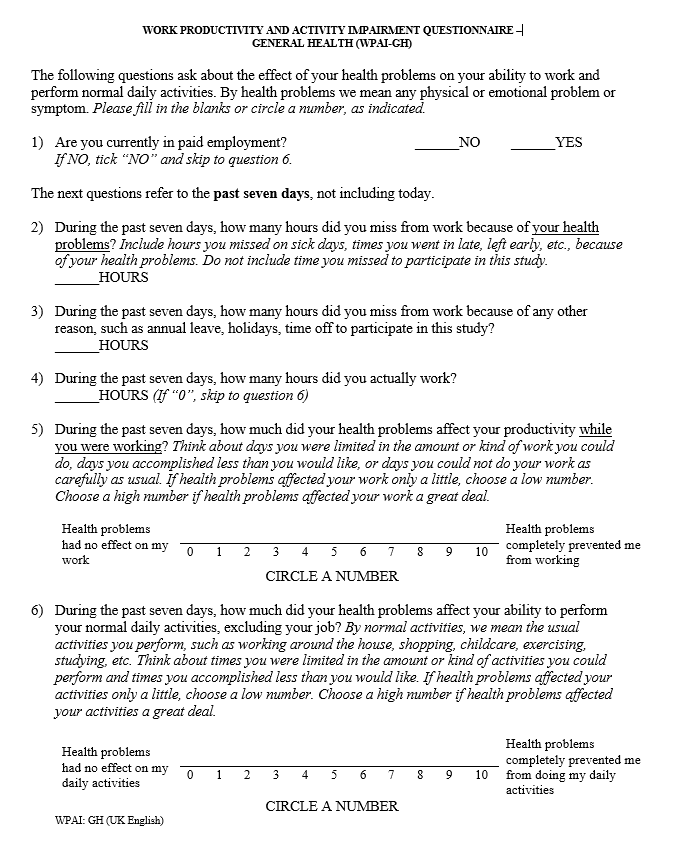


Notes: The study was conducted in Spain and patients completed the validated version in Spanish. The English version is presented here for reference.

**S2** Ad Hoc Questions on employment (only to patients in uncontrolled CS group)

- Were you in employment at the time of your last episode of carcinoid syndrome?

→ If no, were you:

- unemployed

- on temporary medical leave

- on permanent medical leave

- retired

→ If yes, did you go on medical leave during this episode of carcinoid syndrome?

- If yes:

- For how long?
- Who requested it, you or your doctor?

- If no:

- Were there any days that you left work early due to your carcinoid syndrome?
- Were there any days when you did not go to work due to your carcinoid syndrome?

**S3** Use of Investigations

|  | **Usage during study period, no. performed** | | | **Annual cost, euros** | | | | | |
| --- | --- | --- | --- | --- | --- | --- | --- | --- | --- |
| **Investigation** | **Uncontrolled CS**  **n=52** | **Controlled CS**  **n=52** | ***P*-value** | **Uncontrolled CS** | | | **Controlled CS** | | ***P*-value** |
| Morphological and functional Imaging | 4.69 (3.27) | 3.63 (2.11) | 0.3273^b^ | 961.60 (931.03) | | | 887.41 (907.57) | | 0.2317^b^ |
| HRCT | 2.50 (1.88) | 1.96 (1.22) |  | | | | | | |
| Scintigraphy with octreotide | 0.98 (1.15) | 0.81 (1.01) |  |  |  |  |  |  |  |
| MRI | 0.33 (0.65) | 0.52 (0.83) |  |  |  |  |  |  |  |
| Abdominal US | 0.38 (0.77) | 0.17 (0.43) |  |  |  |  |  |  |  |
| Abdominal XR | 0.21 (0.75) | 0.00 (0.00) |  |  |  |  |  |  |  |
| Chest XR | 0.12 (0.70) | 0.00 (0.00) |  |  |  |  |  |  |  |
| PET | 0.08 (0.27) | 0.12 (0.38) |  |  |  |  |  |  |  |
| Angiography | 0.06 (0.31) | 0.00 (0.00) |  |  |  |  |  |  |  |
| Bone scintigraphy | 0.02 (0.14) | 0.00 (0.00) |  |  |  |  |  |  |  |
| Thyroid US | 0.02 (0.14) | 0.00 (0.00) |  |  |  |  |  |  |  |
| MIBG131 scintigraphy | 0.00 (0.00) | 0.04 (0.19) |  |  |  |  |  |  |  |
| Mammography | 0.00 (0.00) | 0.02 (0.14) |  |  |  |  |  |  |  |
| Endoscopy | 0.13 (0.40) | 0.08 (0.27) | 0.7845^b^ | 87.96 (282.67) | | | 49.67 (173.74) | | 0.8803^b^ |
| LGI | 0.08 (0.27) | 0.04 (0.19) |  | | | | | | |
| UGI | 0.04 (0.28) | 0.04 (0.19) |  |  |  |  |  |  |  |
| Endoscopic US | 0.02 (0.14) | 0.00 (0.00) |  |  |  |  |  |  |  |
| Laboratory tests | 16.71 (11.61) | 10.85 (7.86) | 0.0730^b^ | 1967.86 (1721.73) | | | 1257.45 (1056.27) | | 0.1302^b^ |
| Blood test | 14.44 (10.79) | 9.40 (7.15) |  | | | | | | |
| *Chromogranin A* | 3.38 (3.28) | 2.27 (1.91) |  |  |  |  |  |  |  |
| *Insulin* | 0.38 (1.98) | 0.00 (0.00) |  |  |  |  |  |  |  |
| *NSE* | 0.15 (0.80) | 0.13 (0.97) |  |  |  |  |  |  |  |
| *C-peptide* | 0.15 (1.11) | 0.00 (0.00) |  |  |  |  |  |  |  |
| *ProBNP* | 0.04 (0.19) | 0.08 (0.44) |  |  |  |  |  |  |  |
| *Hepatitis serology* | 0.00 (0.00) | 0.02 (0.14) |  |  |  |  |  |  |  |
| Urine test | 2.27 (2.47) | 1.44 (1.67) | 0.0084 ^b^ | | - | - | | - | |
| *Basic test* | 0.44 (1.14) | 0.42 (1.02) |  | | | | | | |
| *5-HIAA* | 1.75 (2.04) | 1.02 (1.35) |  |  |  |  |  |  |  |
| *Serotonin* | 0.02 (0.14) | 0.00 (0.00) |  |  |  |  |  |  |  |
| *5-HTP* | 0.02 (0.14) | 0.00 (0.00) |  |  |  |  |  |  |  |
| *Culture* | 0.04 (0.28) | 0.00 (0.00) |  |  |  |  |  |  |  |
| Biopsy | 0.46 (1.24) | 0.17 (0.43) | 0.0744^b^ | 560.11 (1643.71) | | | 91.73 (228.05) | | 0.0981^b^ |
| Liver | 0.19 (0.69) | 0.17 (0.43) |  | | | | | | |
| Intestine | 0.13 (0.49) | 0.00 (0.00) |  |  |  |  |  |  |  |
| Pancreas | 0.04 (0.28) | 0.00 (0.00) |  |  |  |  |  |  |  |
| Lung | 0.00 (0.00) | 0.00 (0.00) |  |  |  |  |  |  |  |
| Diaphragm | 0.02 (0.14) | 0.00 (0.00) |  |  |  |  |  |  |  |
| Mesorectal | 0.02 (0.14) | 0.00 (0.00) |  |  |  |  |  |  |  |
| Peritoneal | 0.02 (0.14) | 0.00 (0.00) |  |  |  |  |  |  |  |
| Skin | 0.02 (0.14) | 0.00 (0.00) |  |  |  |  |  |  |  |
| Ascitic fluid | 0.02 (0.14) | 0.00 (0.00) |  |  |  |  |  |  |  |
| Immunohistochemistry | 0.58 (1.21) | 0.50 (1.28) | 0.4402^b^ | 28.48 (60.71) | | | 23.80 (60.71) | | 0.4775^b^ |
| Chromogranin | 0.29 (0.70) | 0.15 (0.36) |  | | | | | | |
| Synaptophysin | 0.13 (0.34) | 0.17 (0.38) |  |  |  |  |  |  |  |
| Enolase | 0.04 (0.28) | 0.04 (0.19) |  |  |  |  |  |  |  |
| Insulin | 0.00 (0.00) | 0.00 (0.00) |  |  |  |  |  |  |  |
| CD56 | 0.04 (0.19) | 0.02 (0.14) |  |  |  |  |  |  |  |
| CDX2 | 0.00 (0.00) | 0.02 (0.14) |  |  |  |  |  |  |  |
| KI67 index | 0.08 (0.39) | 0.04 (0.19) |  |  |  |  |  |  |  |
| PAX8 | 0.00 (0.00) | 0.02 (0.14) |  |  |  |  |  |  |  |
| TTF-1 | 0.00 (0.00) | 0.04 (0.19) |  |  |  |  |  |  |  |
| Total investigations |  |  |  | 3606.01 (3133.90) | | | 2310.06 (1829.55) | | 0.0486*^b^ |

Mean (SD) for all parameters.

Abbreviations: 5-HIAA, 5-hydroxyindoleacetic acid; 5-HTP, 5-hydroxy-L-tryptophan; CD56, cluster of differentiation 56; CDX2, caudal type homeobox 2; HRCT, high-resolution computed tomography; LGI, lower gastrointestinal; MIBG131, metaiodobenzylguanidine-131; MRI, magnetic resonance imaging; NSE, neurone-specific enolase; PAX8, paired-box gene 8; PET, positron emission tomography; ProBNP, pro-brain natriuretic peptide; TTF-1, thyroid transcription factor 1; UGI, upper gastrointestinal; US, ultrasound; XR, X-ray.

*statistically significant (P<0.05)

^b^Paired t-test.

**S4** Clinical Characteristics and Management (matched groups).

| **NET characteristics** | **Uncontrolled CS** | **Controlled CS** |
| --- | --- | --- |
| Years since NET diagnosis, median (IQR) | 4.9 (2.7-9.1) | 4.8 (2.0-8.1) |
| Tumour origin, n (%) | | |
| *Lung* | 6 (11.5%) | 6 (11.5%) |
| *Pancreas* | 7 (13.5%) | 7 (13.5%) |
| *Intestine* | 38 (73.1%) | 38 (73.1%) |
| *Other* | 1 (1.9%) | 1 (1.9%) |
| Tumour grade, n (%) |  |  |
| *Grade 1* | 28 (53.8%) | 28 (53.8%) |
| *Grade 2* | 24 (46.2%) | 24 (46.2%) |
| Metastasis site, n (%) |  |  |
| *Liver* | 44 (84.6%) | 49 (94.2%) |
| *Bone* | 6 (11.5%) | 3 (5.8%) |
| *Lung* | 2 (3.8%) | 3 (5.8%) |
| *Brain* | 0 | 0 |
| *Other* | 19 (36.5%) | 15 (28.8%) |
| Investigations used in initial diagnosis, n (%) |  |  |
| Imaging | 41 (78.8%) | 40 (76.9%) |
| *HRCT* | 37 (71.2%) | 36 (69.2%) |
| *Scintigraphy with octreotide* | 29 (55.8%) | 26 (50.0%) |
| *MRI* | 8 (15.4%) | 8 (15.4%) |
| *Scintigraphy with MIBG* | 0 | 0 |
| *PET* | 2 (3.8%) | 7 (13.5%) |
| *Other* | 4 (7.7%) | 3 (5.8%) |
| Biochemistry | 27 (51.9%) | 30 (57.7%) |
| *Serum chromogranin A* | 24 (46.2%) | 28 (53.8%) |
| *Urinary 5-HIAA* | 20 (38.5%) | 22 (42.3%) |
| *Insulin* | 1 (1.9%) | 0 |
| *Other* | 1 (1.9%) | 5 (9.6%) |
| Immunohistochemistry | 30 (57.7%) | 25 (48.1%) |
| *Chromogranin* | 28 (53.8%) | 24 (46.2%) |
| *Synaptophysin* | 23 (44.2%) | 22 (42.3%) |
| *Enolase* | 5 (9.6%) | 2 (3.8%) |
| *Insulin* | 3 (5.8%) | 0 |
| *Other* | 5 (9.6%) | 7 (13.5%) |
| First therapeutic intervention, n (%) |  |  |
| Surgery | 33 (63.5%) | 32 (61.5%) |
| *Partial resection of primary tumour* | 24 (46.2%) | 21 (40.4%) |
| *Complete resection of primary tumour* | 3 (5.8%) | 4 (7.7%) |
| *Bowel resection/ileal anastomosis* | 2 (3.8%) | 4 (7.7%) |
| *Resection of liver metastases* | 2 (3.8%) | 2 (3.8%) |
| *Lymph node resection* | 0 | 1 (1.9%) |
| *Other* | 4 (7.7%) | 5 (9.6%) |
| Pharmacological treatment | 19 (36.5%) | 20 (38.5%) |
| Radiofrequency | 0 | 0 |
| Time since first intervention, years, median (IQR) | 4.5 (2.4-8.7) | 4.4 (1.8-7.9) |
| Time between diagnosis and first intervention, days, median (IQR) | 15.0 (0.0 – 46.0) | 30.5 (0.0 – 7.7.0) |
| Details of last CS episode (uncontrolled group) | | |
| *Time since last CS episode, years, median (IQR)* | 0.9 (0.6-1.8) | - |
| *Duration of last CS episode, months, median (IQR)* | 0.7 (0.1-3.0) | - |
| Pharmacological therapy for NET during study period, n (%) | | |
| *SSA* | 52 (100.0%) | 52 (100.0%) |
| *Targeted therapy* | 18 (34.6%) | 9 (17.3%) |
| *Chemotherapy* | 3 (5.8%) | 2 (3.8%) |
| *Other* | 4 (7.7%) | 3 (5.8%) |
| ***SSA drug*** |  |  |
| *Octreotide* | 26 (50.0%) | 23 (44.2%) |
| *(Octreotide rescue therapy)* | *4 (15.4%)* | 0 |
| *Lanreotide* | 30 (57.7%) | 29 (55.8%) |
| ***Targeted therapy drug*** |  |  |
| *Everolimus* | 16 (30.8%) | 9 (17.3%) |
| *Sunitinib* | 2 (3.8%) | 0 |
| Pharmacological therapy for CS during the study period (uncontrolled group), n (%) | | |
| *SSA* | 52 (100.0%) | NA |
| *Antidiarrheal* | 17 (32.7%) | NA |
| *Antiemetic* | 2 (3.8%) | NA |
| *Antihistamine* | 2 (3.8%) | NA |
| *Glucocorticoid* | 0 | NA |
| *Other^1^* | 5 (9.6%) | NA |
| Signs & symptoms, n (%) | (n=51) | (n=52) |
| Current signs & symptoms (any) | 42 (82.4%) | 12 (23.1%) |
| Diarrhoea | 33 (64.7%) | 7 (13.5%) |
| Skin flushing | 22 (43.1%) | 1 (1.9%) |
| Abdominal pain | 15 (29.4%) | 1 (1.9%) |
| Wheezing | 4 (7.8%) | 0 |
| Dyspnoea | 4 (7.8%) | 1 (1.9%) |
| Intestinal obstruction | 2 (3.9%) | 0 |
| Pellagra | 1 (2.0%) | 0 |
| Other | 2 (3.9%) | 3 (5.8%) |
| Comorbidities, n (%) | (n=52) | (n=52) |
| Hypertension | 13 (25.0%) | 20 (38.5%) |
| Hyperlipidaemia | 7 (13.5%) | 9 (17.3%) |
| Diabetes mellitus | 4 (7.7%) | 8 (15.4%) |
| Cardiovascular disease | 5 (9.6%) | 6 (11.5%) |
| Anxiety/depression | 2 (3.8%) | 6 (11.5%) |
| Valvulopathy | 6 (11.5%) | 2 (3.8%) |
| Other | 7 (13.5%) | 12 (23.1%) |
| Endocrine disorders | 1 (1.9%) | 0 |
| Eye disorders | 0 | 1 (1.9%) |
| Gastrointestinal disorders | 2 (3.8%) | 1 (1.9%) |
| Hepatobiliary disorders | 0 | 1 (1.9%) |
| Infections | 1 (1.9%) | 0 |
| Metabolism and nutrition disorders | 1 (1.9%) | 1 (1.9%) |
| Musculoskeletal and connective tissue disorders | 0 | 2 (3.8%) |
| Neoplasms benign, malignant, or unspecified | 0 | 3 (5.8%) |
| Nervous system disorders | 0 | 2 (3.8%) |
| Renal and urinary disorders | 0 | 2 (3.8%) |
| Reproductive system and breast disorders | 1 (1.9%) | 0 |
| Respiratory, thoracic and mediastinal disorders | 2 (3.8%) | 1 (1.9%) |
| Biochemistry levels, last recorded value |  |  |
| ProBNP, median (IQR) | na | 215.00 (115.0-315.0)  (n=2) |
| 5-HIAA, µmol/24h, median (IQR) | 32.6 (12.5-266.5)  (n=32) | 14.7 (6.6-52.0) (n=31) |
| Chromogranin A, ng/mL, median (IQR) | 1111.5 (261.5-2168.5)  (n=40) | 203.0 (67.0-434.0) (n=45) |
| Last echocardiogram result, n (%) | (n=32) | (n=25) |
| *Normal* | 18 (56.3%) | 15 (60.0%) |
| *Dilatation right cavities* | 7 (21.9%) | 1 (4.0%) |
| *Dilatation left cavities* | 2 (6.3%) | 1 (4.0%) |
| *Tricuspid valve disease* | 8 (25.0%) | 8 (32.0%) |
| *Pulmonary valve disease* | 3 (9.4%) | 1 (4.0%) |
| *Mitral valve disease* | 4 (12.5%) | 3 (12.0%) |
| *Aortic valve disease* | 3 (9.4%) | 2 (8.0%) |
| NYHA class | (n=46) | (n=51) |
| *I-II* | 42 (91.3%) | 51 (100%) |
| *III-IV* | 4 (8.7%) | 0 |
| ECOG status | (n=42) | (n=52) |
| *0-1* | 38 (90.5%) | 51 (98.1%) |
| *2-3* | 4 (9.5%) | 1 (1.9%) |

Notes: ^1^ omeprazole, aspirin, yttrium (90Y), everolimus. 5-HIAA values not available due to not being available in the centre in 5 patients, available but not routine clinical practice in 3 patients and done but not available in 4 patients. For NYHA classification, for deceased patients, the last recorded value in their records was used.

Abbreviations: 5-HIAA, 5-hydroxyindoleacetic acid; CS, carcinoid syndrome; HRCT, high-resolution computed tomography; MRI, magnetic resonance imaging; ECOG, Eastern Cooperative Oncology Group; na, not available; NYHA, New York Heart Association classification; NET, neuroendocrine tumour; P, percentile; PET, positron emission tomography; ProBNP, pro-brain natriuretic peptide; SD, standard deviation; SSA, somatostatin analogue.
